# Detection of Prostate Cancer via IR Spectroscopic Analysis of Urinary Extracellular Vesicles: A Pilot Study

**DOI:** 10.3390/membranes11080591

**Published:** 2021-07-31

**Authors:** Xin-Le Yap, Bayden Wood, Teng-Aik Ong, Jasmine Lim, Bey-Hing Goh, Wai-Leng Lee

**Affiliations:** 1 School of Science, Monash University Malaysia, Jalan Lagoon Selatan, Subang Jaya 47500, Malaysia; xinleyap94@gmail.com; 2 Centre for Biospectroscopy and School of Chemistry, Monash University, Clayton, VIC 3800, Australia; bayden.wood@monash.edu; 3 Department of Surgery, Faculty of Medicine, University of Malaya, Kuala Lumpur 50603, Malaysia; ongta@ummc.edu.my; 4 College of Pharmaceutical Sciences, Zhejiang University, 866 Yuhangtang Road, Hangzhou 310058, China; goh.bey.hing@monash.edu; 5 Biofunctional Molecule Exploratory (BMEX) Research Group, School of Pharmacy, Monash University Malaysia, Subang Jaya 47500, Malaysia

**Keywords:** EVs, exosomes, prostate cancer, FTIR, urine test, diagnosis

## Abstract

Extracellular vesicles (EVs) are membranous nanoparticles naturally released from living cells which can be found in all types of body fluids. Recent studies found that cancer cells secreted EVs containing the unique set of biomolecules, which give rise to a distinctive absorbance spectrum representing its cancer type. In this study, we aimed to detect the medium EVs (200–300 nm) from the urine of prostate cancer patients using Fourier transform infrared (FTIR) spectroscopy and determine their association with cancer progression. EVs extracted from 53 urine samples from patients suspected of prostate cancer were analyzed and their FTIR spectra were preprocessed for analysis. Characterization of morphology, particle size and marker proteins confirmed that EVs were successfully isolated from urine samples. Principal component analysis (PCA) of the EV’s spectra showed the model could discriminate prostate cancer with a sensitivity of 59% and a specificity of 81%. The area under curve (AUC) of FTIR PCA model for prostate cancer detection in the cases with 4–20 ng/mL PSA was 0.7, while the AUC for PSA alone was 0.437, suggesting the analysis of urinary EVs described in this study may offer a novel strategy for the development of a noninvasive additional test for prostate cancer screening.

## 1. Introduction

Prostate cancer, was ranked as the second most frequently diagnosed cancer in males with the estimated age-standardized incidence rate (ASR) of 30.7 per 100,000 and mortality rate of 7.7 per 100,000 worldwide [1]. Prostate-specific antigen (PSA) is currently the most commonly used biomarker in prostate cancer screening, despite its debatable sensitivity and specificity [2]. One of the major drawbacks of this common screening test is an increased PSA level was also observed in patients with benign prostatic hyperplasia (BPH), which commonly occurs in older men [3]. Due to its low specificity in prostate cancer detection, PSA test has led to an increasing number of unnecessary prostate biopsies which involve an invasive procedure causing discomfort and stress to patients [4]. With the emergence of patient-centered care concept in current modern medicine, researchers are keen and working hard in identifying a biomarkers with higher sensitivity and specificity for effective early detection. In addition, it would be even greater if the biomarkers could be detected from human excretions noninvasively.

Extracellular vesicles (EVs) are a heterogeneous group of nano-sized, membrane-bound vesicles shed from the plasma membrane that can transport a complex cargo, including proteins, lipids and nucleic acids, between cells [5,6]. EVs have raised research interest lately because they were found to be associated with malignancy as increased secretion of EVs has been observed in cancer cells. Some of the EVs in particular were linked to resistance to cancer treatment, contributing to the process of angiogenesis, an important attribute of a malignant tumor [7]. In general, several proteins [8,9], mRNAs [10], microRNAs [11,12] and lipids [13,14] present in prostate cancer-derived EVs are potential biomarkers for prostate cancer. Compared to other body fluids, such as blood and semen, urine sample is highly preferred for prostate cancer detection as the approach is noninvasive and easily obtained from patients. The composition of urine reflects the condition of the urogenital system directly [8]. Therefore, urinary EVs have emerged as potential biomarkers for prostate cancer and numerous studies have been carried out to investigate its role in disease progression [8,9,10,11,12,15,16].

In the current study, attenuated total reflection Fourier-transform infrared (ATR-FTIR) spectroscopy was attempted for its potential application to investigate EVs isolated from urine samples in a more focused manner. Although the technique has been applied to differentiate between normal benign and prostate cancer biopsies since the 1990s [17], its accuracy is rather a big question as complex tissue does not really provide substantial evidence for diagnostic purposes. Nevertheless, FTIR is considered a rather robust and cost-effective approach for analyzing any molecular composition of samples. Deciphering the biological moieties present in EVs using FTIR might shed light for more accurate detection [18]. The improvement in the precision and accuracy of detection might further substantiate its application in future for disease staging purposes. The whole idea is built on core fundamental scientific understanding, whereas a cell tends to release a rather distinctive set of biochemical composition for signaling or regulatory alteration in different stages of disease progression. It was well proven that different EVs composed distinctive biological moieties at different stages of cancer [8,9,10,11,12]. By tapping into this unique behavior, a rather focused screening of EVs derived from various stages of cancer using ATR-FTIR might enable us to generate sets of unique fingerprint-like information to distinguish one from another [19]. In view of the great advantages of ATR-FTIR use as a point-of-care diagnostic test in a clinical setting—time saving, portable, inexpensive, no chemical stains or reagents are required for the biochemical analysis of the sample [20], there would be no hesitation for us to streamline and improve the sensitivity and accuracy of the said method. To address these, urine-isolated EVs were derived from normal individuals and prostate cancer patients whilst spectra of EVs was generated for these two distinctive groups using ATR-FTIR. A further insight analysis into unique spectroscopic finger-print information can potentially be utilized in the development of a noninvasive urine test for the early detection of prostate cancer.

## 2. Materials and Methods

### 2.1. Study Population

A total of 53 treatment-naïve patients suspected of prostate cancer who underwent transrectal ultrasound (TRUS)-guided prostate biopsy at University of Malaya Medical Centre were recruited between 2016 and 2019. Serum and urine samples were collected for this study prior to initiation of TRUS-guided prostate biopsy. Specimens were divided into two groups including 31 non-cancerous and 22 cancerous based on the TRUS-guided prostate biopsy results. Twenty-two cancerous samples were then further grouped into three Gleason score (GS) groups: 4 samples in GS < 7, 5 samples in GS = 7 and 13 samples in GS > 7. All participants provided written informed consents. The study protocol was approved by the Medical Research Ethics Committee of University of Malaya Medical Centre (MREC ID NO: 2017728-5442) and Monash University Human Research Ethics Committee (Project ID: 13232).

### 2.2. Isolation of EVs

Urine samples were preserved in sodium azide (20 mM) and kept at −80 °C. Urinary EVs were isolated according to our previous optimized method [21]. Briefly, urine samples were transferred to 4 °C fridge a day before EV isolation. Samples were vortexed for 30 s before centrifuging at 400× *g* and 15,500× *g* for 20 min, respectively (Eppendorf 5804 Centrifuge). The supernatant was then centrifuged at 200,000× *g* for 90 min at 18 °C (Thermo Scientific, Rotor Type T-865, Sorvall WX 100+ Ultracentrifuge, Watertown, MA, USA). The supernatant was discarded and the pellet was resuspended in phosphate-buffered saline (PBS) containing 0.5 mL of 1 M DTT. Samples were centrifuged again at 200,000× *g* for 70 min at 25 °C using a TH-641 rotor. The supernatant was discarded; pellet containing EVs was resuspended in 100 μL PBS and stored in −80 °C until further analysis. The protein content of the isolated EVs was determined using Bradford assays.

### 2.3. Characterization of EVs

#### 2.3.1. Transmission Electron Microscopy (TEM)

Freshly prepared urinary EVs were fixed in equal volume of 4% paraformaldehyde. A 5 μL sample of fixed EVs were deposited on a formvar coated grid (copper 300 mesh), allowed absorbance in a dry environment for 20 min at room temperature. The grid was then washed with PBS, dried using filter paper, negatively stained using 5 µL of 1% (*w*/*v*) uranyl acetate for 10 min. TEM images of the stained EVs were captured using FEI Tecnai G2 20 S-Twin transmission electron microscope at 200 kV.

#### 2.3.2. Particle Size Analysis (Zetasizer)

Thawed EV sample was diluted to 0.1 μg/500 μL per tube and filtered using a 0.45 μm Ultrafree-MC Centrifugal Filter (Merck, Darmstadt, Germany). The samples were then analyzed using Zetasizer Nano ZS (Malvern Instruments, Amesbury, UK). Each experimental run was performed in triplicate.

#### 2.3.3. Immunoblotting

Loading buffer was added to 10 µg of EV protein sample and boiled at 95 °C for 5 min, separated in a 12% SDS-polyacrylamide gel (SDS-PAGE) and electrophoretic transferred to nitrocellulose membranes. Membranes were blocked in 2.5% nonfat dry milk in 1x TBS-T (0.5% Tween-20), at room temperature and incubated with the following primary antibodies: rabbit anti-CD9 (1:2000) (sc-9148; Santa Cruz Biotechnology Inc., Santa Cruz, CA, USA), rabbit anti-CD10 (1:3000) (GTX111680; Genetex, Irvine, CA, USA), mouse anti-HSC70 (1:2000) (sc-7298; Santa Cruz Biotechnology, Inc.), mouse anti-TSG101 (1:1000) (sc-7964; Santa Cruz Biotechnology, Inc.). Membranes were then washed with 1x TBS-T (0.5% Tween-20). The secondary antibodies used were the mouse IgGκ BP conjugated to HRP (1:2000) (sc-516102; Santa Cruz Biotechnology, Inc.); and the mouse anti-rabbit IgG-HRP (1:2000) (sc-2357; Santa Cruz Biotechnology, Inc.). Secondary antibodies were incubated for 2 h, with agitation at room temperature. Following TBS-T washes, protein bands were detected using the chemiluminescence reagent WesternBright ECL HRP substrate (Advansta Inc., San Jose, CA, USA.) and images acquired with G:BOX Chemi XX9 gel imaging system (Syngene, Cambridge, UK).

### 2.4. Attenuated Total Reflection-Fourier Transform Infrared Spectroscopy (ATR-FTIR)

The thawed EV sample was diluted to 0.1 μg/500 μL per tube and filtered using a 0.45 µm Ultrafree-MC Centrifugal Filter (Merck). ATR-FTIR spectra of the samples were collected using a Perkin Elmer Spectrum Two FTIR Spectrometer. For each spectrum 32 interferograms were co-added at a spectral resolution of 4 cm^−1^ in the 4000 to 400 cm^−1^ range. Background scans were obtained from the diamond ATR crystal internal reflection element after cleaning. The spectrum of ultrapure water and PBS were acquired. The crystal was cleaned again then loaded with 2 µL of filtered sample (~0.4 ng of EV protein). The solvent was removed using a blow dryer. A total of 53 spectra (31 control and 22 cases) were collected and employed for the data analysis. ATR baseline correction and normalization were performed after the spectra were acquired.

### 2.5. Statistical Analysis

The Unscrambler X 10.5.1 was used to preprocess the spectra, which included baseline correction, unit vector normalization, first derivatives (Savitzky-Golay, 21 smoothing points) and standard normal variate (SNV) transformation. The spectral region used was 1400–800 cm^−1^. Using the same software, principal component analysis (PCA) and partial least square analysis (PLS) using 7 PCs with cross validation were performed. The condition of the patient (cancerous and non-cancerous) and Gleason score (GS < 7, GS = 7, GS > 7) was used as the function to find differences within the spectral data set. The average points (centroids) in PCA score plots were generated according to the mean of all samples in the respective clusters. The Euclidean distances between the centroid and all points in the cluster were derived and scored. The Euclidean models built on the PCA score plots for both cancerous vs. non-cancerous and Gleason clustering were used to measure the sensitivity and specificity of FTIR analysis of urinary EVs in prostate cancer detection and classification. Further, the discrimination abilities of FTIR analysis of urinary EVs and PSA above a specific threshold were determined with the area under the receiver operating characteristic curve (AUC) using SPSS for Windows version 21.0 (SPSS Inc., Chicago, IL, USA).

## 3. Results

### 3.1. Characterization of Urinary EVs

Table 1 summarizes the histological data including PSA levels for prostate cancer patients involved in this study. The PSA level of patients with negative prostate biopsy results were recorded in Appendix A Table A1. The cases (*n* = 22) comprised a representative set of prostate cancer-derived EVs from different disease stages. Four samples were grouped as GS < 7, five samples were grouped as GS = 7 and thirteen samples were grouped as GS > 7. Prostate sample 53 was clinically diagnosed based on computerized tomography (CT) scan and bone scan imaging results. The median PSA levels were 8.49 ng/mL and 28.94 ng/mL for control (*n* = 31) and cases, respectively. EVs were successfully isolated from urine samples collected prior to TRUS biopsy. The characteristics of the isolated EVs were examined using TEM, particle size analysis by Zetasizer and immunoblotting.

**Figure 1:**
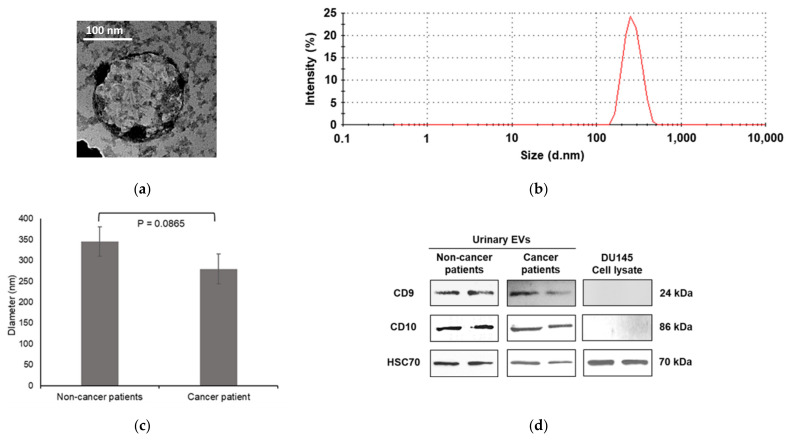
Characterization of urinary EVs: (**a**) transmission electron micrographs; (**b**) intensity plot for EV size distribution analyzed using Zetasizer; (**c**) comparison of average diameter between urinary EVs of healthy individuals and prostate cancer patients (Student *t*-test, *n* = 15); (**d**) immunoblot analysis of EV markers in urinary EVs and DU145 cell lysate.

Figure 1a shows a representative TEM image of EVs isolated from urine patients. The EV was round and in the form of a membraned vesicle. Zetasizer was then employed to measure the sizes of the isolated urinary EVs. Figure 1b demonstrates the representative size distribution by intensity plot analyzed using Zetasizer, indicating an average size between 248 to 268 nm for the majority of urinary EVs. The mean diameter of the EVs isolated from the urine of non-cancer patients was found to be slightly higher when compared to that of prostate cancer patients. However, the difference between the two groups was statistically insignificant (*p* = 0.0865, Figure 1c). Immunoblotting (Figure 1d) confirmed the presence of EV markers in the nanovesicles isolated from the urine samples of both the cancer and non-cancer patients, including CD10, CD9 and HSC70.

### 3.2. FTIR Analysis of Urinary EVs

**Figure 2:**
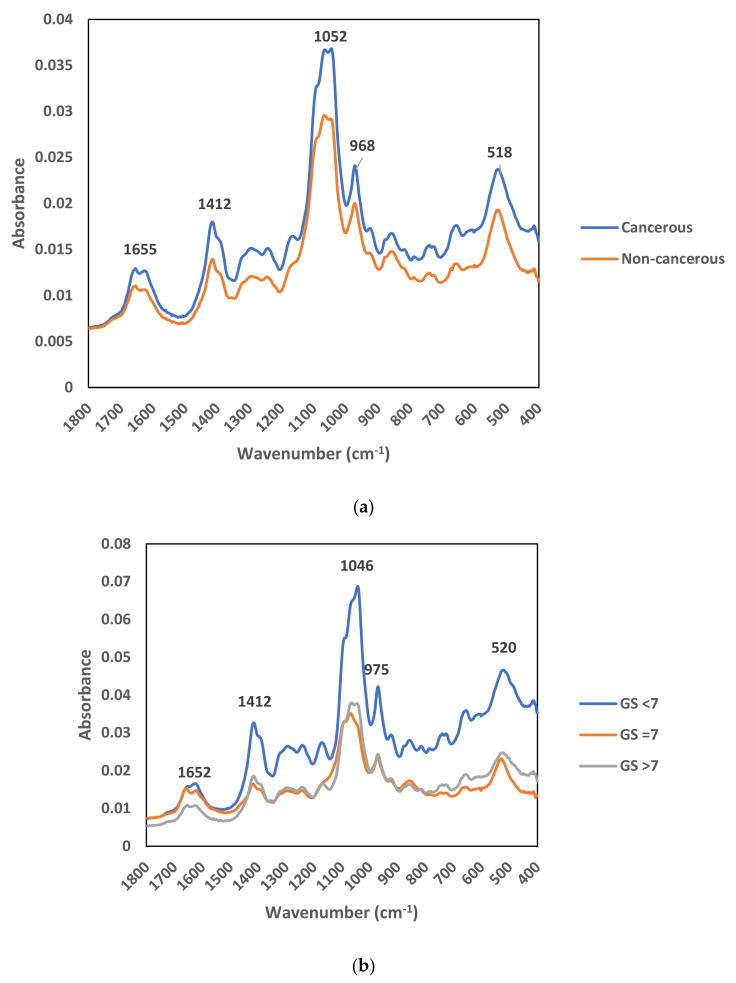
Average raw FTIR spectra of urinary EVs: (**a**) cancerous vs. non-cancerous EVs; (**b**) Gleason score groups (GS < 7, GS = 7, and GS > 7).

The average raw spectrum of the comparison between non-cancerous and cancerous EVs and the comparison between three Gleason score groups are shown in Figure 2a,b, respectively. Absorbance of the spectrum for the cancerous EVs was higher at a few peaks as compared to non-cancerous EVs, indicating a difference between these two groups (Figure 2a). The spectrum of absorbance for GS < 7 EVs was higher as compared to GS = 7 and GS > 7 groups, indicating a difference between the early stage and the later stages of the cancers (Figure 2b).

### 3.3. Principle Component Analysis (PCA)

**Figure A1:**
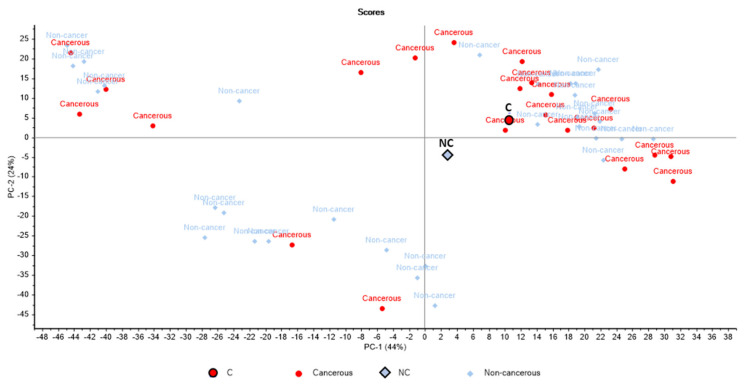
Principal component analysis of the subsets of EVs preprocessed by Unscrambler X. Score plot of the first and second PCs, with the corresponding percentage of explained variance in parentheses. (C: average spectrum of cancerous samples, NC: average spectrum of non-cancerous samples).

**Figure A2:**
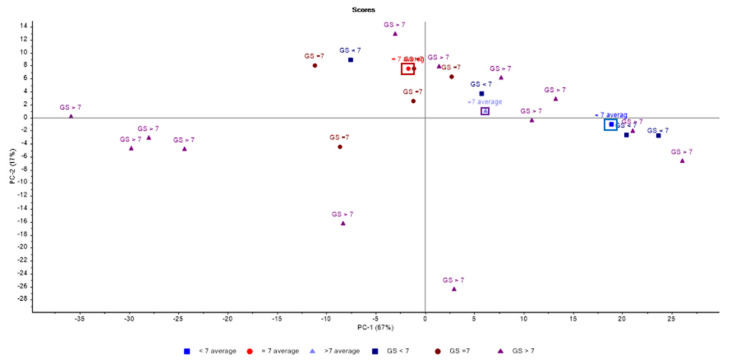
Principal component analysis of the subsets of prostate cancer associated urinary EVs preprocessed by Unscrambler X. Score plot of the first and second PCs, with the corresponding percentage of explained variance in parentheses.

An unsupervised test was carried out to assess the accuracy of the grading model using IR spectra from 53 urinary EV samples. The score plot derived from PCA analysis of processed IR spectra (Figure A1) was used in training dataset development (average spectra) to predefine the groups of cancerous and non-cancerous samples. The minimum Euclidean distances between the spectra of urinary EV samples to respective average spectrum of the two predefined groups were used to predict the actual cancer condition of the samples. The confusion matrix shown in Table 2 summarizes the capability of the Euclidean model built on the PCA score plot for cancerous vs. non-cancerous samples (Figure A1). Among the 53 individuals detected with the suspicious PSA levels, 31 were diagnosed with no prostate cancer after the TRUS biopsy (Table A1). Some 25 out of 31 urinary EV samples obtained from these prostate cancer-free individuals were classified in the non-cancerous group based on Figure A1 PCA-Euclidean analysis, suggesting this model would have a specificity of 81% in prostate cancer discrimination, while 13 out of 22 samples from the diagnosed prostate cancer patients were classified in the cancerous group implies the model could achieve 59% of sensitivity in cancer detection. Another PCA analysis was carried out to assess the performance of the Gleason grading model using the IR spectra of 22 cancerous urinary EV samples. The resulting score plot shown in Figure A2 was used to build the Euclidean model for predicting the Gleason score of the prostate cancer biopsy. There were 13 patients diagnosed with late stage prostate tumors (Gleason score > 7) while fewer (4 patients) were diagnosed with early stage (Gleason score < 7) (Table 1). Based on PCA-Euclidean model in Figure A2, only 31% and 50% of the respective urinary EVs were classified in the actual scoring groups. However, all of the EVs samples collected from prostate cancer patients with Gleason score = 7 were correctly predicted using the model (Table 3).

### 3.4. Spectral Peaks Responsible for Prostate Cancer Discrimination

The IR spectra of the urinary EVs provided essential information about the biomolecular composition of the membranous vesicles. Our PCA analysis found several spectral peaks significantly contributed to the discrimination between cancerous and non-cancerous samples as well as the different stages of prostate cancers. These spectral peaks were reported as the absorbance bands of various bond vibrations whose functional groups are involved in the formation of different biomolecules [22]. The biomolecular assignments observed in this study were listed in Table 4. In addition, partial least square (PLS) analysis was performed on these absorbance bands to identify the key contributors responsible for the cancer classification. The resulting weighted regression coefficients suggest the absorbance bands with the wavenumbers of 988, 1121 and 1081 cm^−^^1^, which are related to bond vibration in RNA, contribute the most to the discrimination between cancerous and non-cancerous samples (Figure A4a). On the other hand, the absorbance bands (1050, 1057 and 1071 cm^−1^) of CO bond vibration in carbohydrate and DNA demonstrate the highest values of weighted regression coefficients, resembling the key contributors to the Gleason discrimination in prostate cancer (Figure A4b).

### 3.5. Accuray of FTIR Analysis of Urinary EVs in Prostate Cancer Detection

In Section 3.3, the sensitivity and the specificity of the FTIR analysis of urinary EVs in prostate cancer detection were measured using the Euclidean model built on PCA score plots (Figure A1). Furthermore, the accuracy of this model was examined using a receiver operating characteristic (ROC) curve. A wide range of PSA levels (4–1200 ng/mL) was detected in all individuals with or without prostate cancer participating in TRUS biopsy (Table 1 and Table A1). Therefore, the area under the curve (AUC) for both PSA test and FTIR-PCA analysis were measured and compared (Figure A5). Besides considering the ROC for the samples collected from all the participants, the AUC of the subsets with low (4–10 ng/mL) to medium (4–20 ng/mL) levels of PSA were also measured (Table 5). The results showed the FTIR analysis of urinary EVs shared a similar AUC (0.723) with the PSA test (0.724) which may be interpreted as both methods have ~72% of probability of correctly distinguish between prostate cancer and non-cancerous cases. However, the probabilities of low to medium levels of PSA in a blood test for detecting the cancer were rather low (44–45%) when compared to FTIR analysis of urinary EVs which have 63–70% chance of diagnosing prostate cancer in the individuals detected with PSA in the low range of 4 ng/mL up to 10 or 20 ng/mL.

## 4. Discussion

The ATR-FTIR spectroscopic approach has been used to differentiate between normal benign and prostate cancer biopsies since the 1990s. A previous study utilized ATR-FTIR to discriminate DNA samples between healthy and cancer-suffering individuals achieving a rather substantial reliability in both sensitivity and specificity [17]. In addition, this technique was also used to monitor the radiotherapeutic response in prostate cancer patients, showing differences between minimal versus severe acute and late toxicity of the therapy with sensitivity and specificity ranging from 80 to 99% [23]. However, these protocols were still unable to address the invasiveness issue and physiological stress being imposed on patients during the biopsies. In order to unleash ATR-FTIR’s full potential and any future deployment as a point-of-care diagnostic test in a clinic setting for prostate cancer, the refinement of the analytic approach is the key to success.

As part of our continuous effort in the establishment and refinement of ATR-FTIR method use in prostate cancer diagnosis, the study was focused on the spectra analysis of the EVs in urine. It is mainly due to the fact that EVs are known to be excreted in urine and the anatomically close proximity of prostate cancer tissue granted a higher chance of intact prostate cancer EVs being found in urine. By tapping into the fundamental understanding on spectra absorption principle—a difference in biochemical compositions allows the generation of distinctive spectra based on the absorption bands specific to the chemical species present in a measured sample [22], the differences could be captured for an effective differentiation effort of each other, for instance between the normal individuals and prostate cancer-bearing patients. The IR spectra collected in our study implied the most pronounced changes in cancerous samples were observed in the fingerprint region, specifically at the wavenumbers from 968 to 1419 cm^−1^ (Figure 2). Our findings are in line with the existing knowledge of where the biochemical differences between tumor and normal cells were often detected between 1800 and 950 cm^−1^, a wavenumber range known as the “fingerprint region” [24].

**Figure A3:**
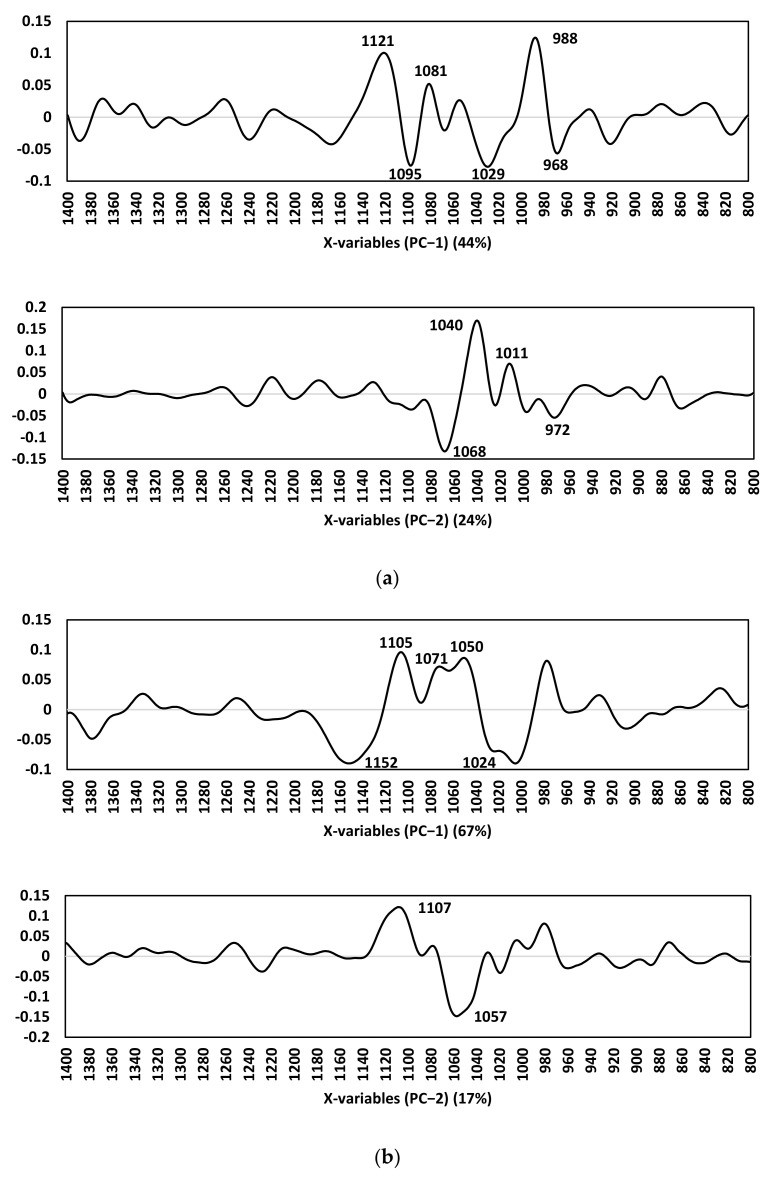
Loading plots of the first and second PCs. (**a**) Cancerous and non-cancerous groupings. (**b**) Gleason score groupings.

**Figure A4:**
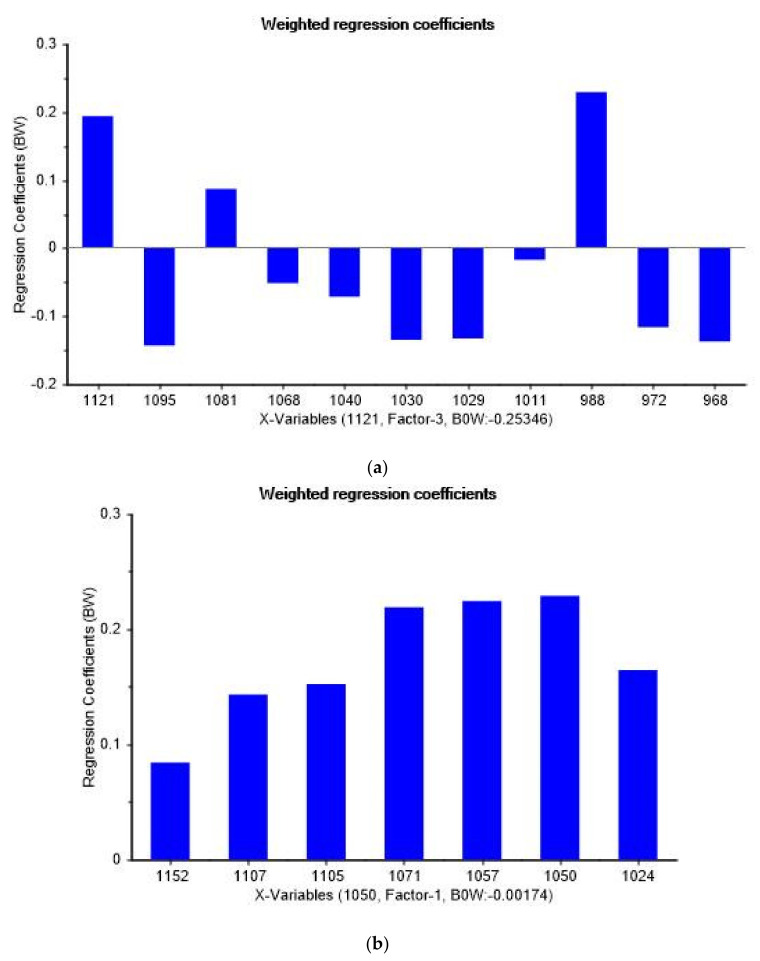
Weighted regression coefficients generated using partial least squares (PLS) analysis for the major spectral peaks responsible for (**a**) the cancer discrimination, and (**b**) the Gleason score discrimination.

**Figure A5:**
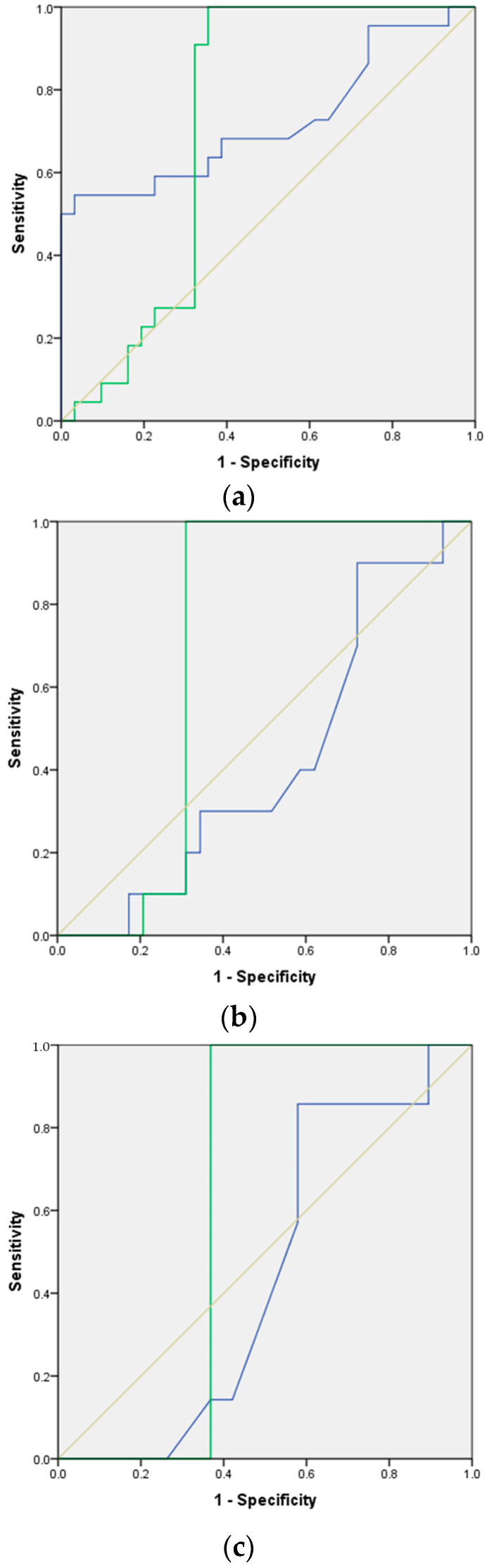
Area under the receiver operating characteristic (ROC) curve (AUC). Range of PSA Levels: (**a**) 4–1200 ng/mL; (**b**) 4–20 ng/mL; (**c**) 4–10 ng/mL.

Principal component analysis (PCA) was used in the following study to reduce the dimensionality of the complex datasets extracted from the IR spectra of urinary EVs. Score plots that were generated based on the first two principal components (PC1 and PC2) interpreted how the different sets of spectral peaks contribute to the discrimination of the status of prostate cancer (Figure A3). Euclidean distance models built on these score plots provides preliminary evidence for the use of our FTIR PCA model in discrimination of prostate cancer as well as diagnosis of different stages of the cancer progression. From the model, twelve wavenumber values were found to be responsible for the cancer and non-cancer discrimination while seven of the wavenumber values were correlated to the differences between Gleason score groupings (Table 4). The most significant spectral differences (988, 1121 and 1081 cm^−1^) between the cancer and non-cancer groups occur in the RNA and phosphodiester regions. The 1081 cm^−1^ band is especially important as phosphodiester stretching modes from nucleic acids and suggests an increase in the nucleic acids in the malignant tissues resulting from uncontrolled cell cycle progression of cancer cells. The significant shifted peaks at ~1080 cm^−1^ were also observed in cancers including colon, ovaries, skin and cervix [24]. The similar peak shift observed in our study implies the EVs in the urine of prostate cancer patients may carry more DNA/RNA originated from malignant tissues as an indication of tumor growth in the body.

For Gleason score discrimination, spectral differences mainly occur at the glycogen, DNA, polysaccharides and carbohydrate region (1200–950 cm^−1^), among which 1050 cm^−1^ was identified as the most important contributor in discriminating the different stages of prostate cancer in our PLS analysis. This absorbance indicates the bond vibration in glycogen/carbohydrates [22]. The glycogen levels in the sample play a crucial role in determining the stage of malignancy as a lower glycogen level indicates that the EV-secreting cells are proceeding through their cell cycle thus exhibiting a relatively higher metabolic activity, which is supported by studies carried out by Gazi et al. [25]. Glycosylation is the reaction in which a carbohydrate (oligosaccharide) is attached to a biomolecule. The EV membrane consists of abundant glycoproteins and surface glycosylation of urinary EVs was reported to demonstrate disease-specific modifications [26]. Lately, a significant level of glycoproteins was identified in the EVs of breast cancer patients [27]. Together, these findings indicate that the detection of carbohydrate/glycogen in urinary EVs using FTIR could be a crucial attribute of our analysis which could discriminate against the different stages of prostate cancers.

**Figure A6:**
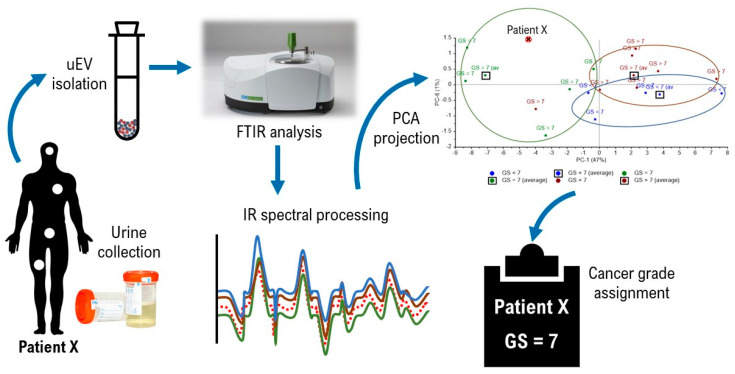
Suggest workflow for FTIR analysis of urinary EVs and derivation of diagnostic classifier for prostate cancer detection using PCA score plot.

Among all 53 individuals detected with elevated PSA levels in this study, 31 with PSA 4.19–23.52 ng/mL did not have prostate cancer after undergoing TRUS biopsy, reflecting the reliability issue of PSA being a widely used diagnostic tool with a cut off value at 4 ng/mL. There has been substantial debate in the past years regarding both the high false positive and false negative rates of PSA, particularly in the range of 4–10 ng/mL which is now known as the gray area in prostate cancer detection [4]. Interestingly, the AUC derived from our FTIR PCA model indicates urinary EVs have up to a 70% chance of detecting prostate cancer correctly in those individuals with PSA 4–20 ng/mL. Conversely, the AUC of the PSA model alone was 44% suggesting additional clinical parameters are required to enhance its accuracy. Since ATR-FTIR is a simple, rapid, accurate, inexpensive, noninvasive method [28], the FTIR analysis of urinary EVs coupled with the multivariate (PCA) model (Appendix B Figure A6) would be a potential noninvasive alternative for the cases with indecisive outcome of PSA test.

## 5. Conclusions

The existing exploratory study evidenced by targeting EVs in urine, the sensitivity and reliability of FTIR use as a potential diagnosis method for prostate cancer detection has been improved significantly. An obvious spectral discrimination of EV was found between cancerous and non-cancerous cases. The crucial difference in Gleason grading was observed, lending further support for the current notion. The trial carried out proved the current model concept exhibits a higher accuracy in detecting prostate cancer for cases with a suspiciously low level of PSA (4–10 ng/mL) compared to the PSA blood test. Therefore, current noninvasive screening approach which focuses on prostate cancer-derived EVs in urine holds great potential to be developed as a complementary test for future screening of prostate cancer to avoid unnecessary painful biopsies.

## Figures and Tables

**Table 1:** Summary of patient information for prostate cancer specimens.

Patient	Histologic Gleason Grade	Histologic Gleason Score	Actual Gleason Score Grouping	Predicted Gleason Score Grouping	Tumor Stage	PSA (ng/mL)
32	5 + 4	9	GS > 7	GS < 7	T4N1M1b	806
33	3 + 3	6	GS < 7	GS < 7	T2aN0M0	7
34	3 + 4	7	GS = 7	GS = 7	T2cN0M1b	73
35	4 + 4	8	GS > 7	GS > 7	T3aN0M0	7
36	3 + 4	7	GS = 7	GS = 7	T2cNxMx	25
37	3 + 4	7	GS = 7	GS = 7	T2aN0M0	4
38	4 + 4	8	GS > 7	GS < 7	T2bN0M0	121
39	3 + 4	7	GS = 7	GS = 7	T1aNxM0	13
40	3 + 4	7	GS = 7	GS = 7	T2aN0Mx	6
41	3 + 3	6	GS < 7	GS > 7	T2aN0M0	10
42	3 + 3	6	GS < 7	GS < 7	T2cN0M0	33
43	5 + 4	9	GS > 7	GS < 7	T3bN0M0	7
44	4 + 5	9	GS > 7	GS = 7	T4N1M1b	7
45	4 + 4	8	GS > 7	GS > 7	T2cN0M1b	188
46	5 + 4	9	GS > 7	GS = 7	TxNxM1b	11
47	3 + 3	6	GS < 7	GS = 7	T2cN0M0	8
48	5 + 5	10	GS > 7	GS > 7	T4N1M1c	231
49	4 + 4	8	GS > 7	GS > 7	TxNxM1b	1178
50	4 + 5	9	GS > 7	GS = 7	TxNxM1b	260
51	5 + 5	10	GS > 7	GS = 7	T4N1M1b	256
52	4 + 4	8	GS > 7	GS = 7	TxNxM1b	1178
53 ^1^	n.a. ^2^	n.a. ^2^	GS > 7	GS = 7	TxNxM1b	101

^1^ This patient was clinically diagnosed based on computerized tomography (CT) and bone scan imaging results. ^2^ Abbreviation: n.a., not applicable.

**Table 2:** Confusion matrix for classification of cancerous vs. non-cancerous using FTIR analysis of urinary EVs.

	Status	Predicted Group Membership		Total
		Non-Cancerous	Cancerous	
Count	Non-cancerous	25	6	31
	Cancerous	9	13	22
%	Non-cancerous	81	19	100
	Cancerous	41	59	100

**Table 3:** Confusion matrix for classification of different stages of prostate cancer using FTIR analysis of urinary EVs.

	Gleason Score	Predicted Group Membership			Total
		GS < 7	GS = 7	GS > 7	
Count	GS < 7	2	1	1	4
	GS = 7	0	5	0	5
	GS > 7	3	6	4	13
%	GS < 7	50	25	25	100
	GS = 7	0	100	0	100
	GS > 7	23	46	31	100

**Table 4:** Major spectral peaks responsible for the cancer discrimination and proposed biomolecular assignments for (**a**) PC-1 and (**b**) for PC-2. Major spectral peaks responsible for the Gleason score discrimination and proposed biomolecular assignments for (**c**) PC-1 and (**d**) for PC-2 (Figure A3).

**(a)**	**Direction**	**Wavenumber (cm^−1^)**	Proposed Biomolecular Assignment *
	+ve	988	RNA stretching, ring bending of uracil
	+ve	1081	Symmetric phosphate stretching modes ʋ(PO^2−^) symmetric stretching of phosphodiesters Phosphate I in RNA One of the triad peaks of nucleic acids
	+ve	1121	Symmetric phosphodiester stretching band, RNA Shoulder of 1121 cm^−1^ band due to RNA
	−ve	968	C-N-C stretch: nucleic acids
	−ve	1029/30	O-CH_3_ stretching of methoxy groups
	−ve	1095	Stretching PO^2−^ symmetric
**(b)**	**Direction**	**Wavenumber (cm^−1^)**	Proposed Biomolecular Assignment *
	+ve	1011	CHα,α’ out-of-plane bending and C_α_ = C_α’_ torsion
	+ve	1040	Stretching CO ribose
	−ve	972	OCH_3_ (polysaccharides, pectin)
	−ve	1068	Stretching CO ribose
**(c)**	**Direction**	**Wavenumber (cm^−1^)**	Proposed Biomolecular Assignment *
	+ve	1050	ʋs CO-O-C C-O stretching coupled with C-O bending of the C-OH of carbohydrates Glycogen
	+ve	1071	Phosphate I band for 2 different CO vibrations of deoxyribose in DNA in disordering structure
	+ve	1105	Carbohydrates
	−ve	1024	Glycogen (CO stretch associated with glycogen)
	−ve	1152	Glycogen absorption due to CO and CC stretching, and COH deformation motions
**(d)**	**Direction**	**Wavenumber (cm^−1^)**	Proposed Biomolecular Assignment *
	+ve	1107	ʋ(CO), ʋ(CC), ring (polysaccharides, pectin)
	−ve	1057	Stretching CO deoxyribose

* Reference: Talari et al., 2017 [22].

**Table 5:** Area under a receiver operating characteristic (ROC) curve for comparison of PSA and FTIR analysis of urinary EVs in discriminating between the cancerous and non-cancerous cases.

Range of PSA Levels [ng/mL]	Area Under Curve (AUC)	
	PSA	FTIR Analysis of Urinary EVs
4–1200	0.724	0.723
4–20	0.437	0.700
4–10	0.447	0.632

**Figure A7:**
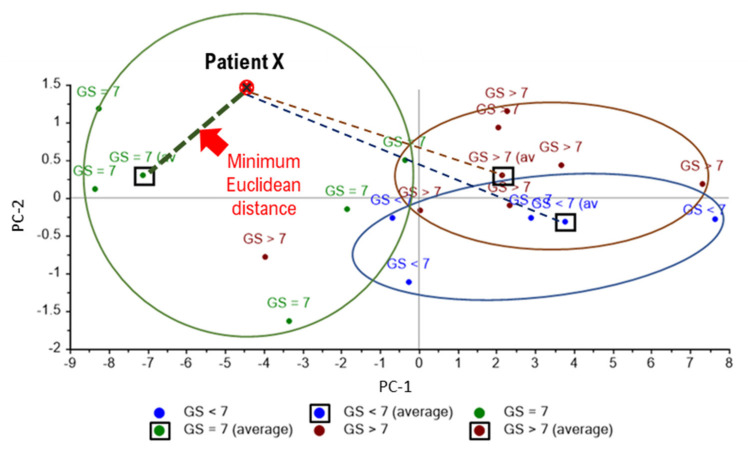
Example of prostate cancer classification using FTIR PCA model established using urinary EVs.

## Data Availability

The data presented in this study are available on request from the corresponding authors. The data are not publicly available due to its ethical concerns that could compromise the privacy of research participants.

## Appendix A

**Table A1:** PSA levels in patients with negative TRUS biopsy results.

**Patient**	**PSA (ng/mL)**	**Patient**	**PSA (ng/mL)**	**Patient**	**PSA (ng/mL)**
1	7	11	19.69	21	12
2	15	12	8.27	22	8
3	6	13	30	23	10
4	8	14	11.95	24	12
5	14	15	4.3	25	6
6	5	16	5.77	26	12
7	17	17	20	27	9
8	11	18	6.8	28	24
9	8	19	6	29	4
10	9	20	6.16	30	8
				31	7

## Appendix B. Suggested Workflow for FTIR PCA Model of Urinary EV Analysis

1 . **Isolate extracellular vesicles (EVs) from urine samples**

Materials

- Urine preserved in sodium azide (20 mM)
- Phosphate-buffered saline (PBS)
- Dithiothreitol (DTT) (1 M) in ultrapure water
- 50-mL polypropylene centrifuge tubes
- Ultracentrifuge and fixed-angle or swinging-bucket rotor
- Polyallomer tubes or polycarbonate bottles

Procedure

Preserved urine samples should be stored at −80 °C and to be moved to 4 °C storage 1 day prior to urinary EVs isolation.

1. Vortex the urine samples for 30 s each. Transfer the fluid into 50-mL tubes.
2. Centrifuge the urine samples at 400× *g* and 15,500× *g* for 20 min respectively.
3. Carefully transfer the supernatant to ultracentrifuge tubes and spin at 200,000× *g* using a fixed-angle rotor for 90 min at 18 °C.
4. Discard the supernatant and resuspend the pellet using 0.5 mL of 1M DTT and 1.5 mL of PBS.
5. Incubate the suspensions at 37 °C for 10 min.
6. Top up the suspensions with PBS and centrifuge the samples at 200,000× *g* using a swinging-bucket rotor for 70 min at 25 °C.
7. Pour away the supernatant and resuspend the pellet using 100 µL of PBS.
8. Quantify the protein content of resuspended EVs using Bradford assay.
9. Store at −80 °C until further analysis.

2 . **Acquire infrared (IR) spectra of urinary EVs using attenuated total reflection-Fourier transform infrared (ATR-FTIR) spectroscopy**

Materials

- 0.45 µm Ultrafree-MC Centrifugal Filter (Merck)
- Phosphate-buffered saline (PBS)
- Ultrapure water
- Blow dryer
- FTIR spectrometer

Procedure

1. Dilute thawed urinary EVs to 0.1 μg/500 μL per tube and filter the samples using a 0.45 µm Ultrafree-MC Centrifugal Filter (Merck).
2. Set the settings of the FTIR machine to 32 scans, 4 cm^−1^ resolution and wavelength from 4000 cm^−1^ to 400 cm^−1^.
3. Acquire a background spectrum.
4. Acquire the spectrum of ultrapure water and PBS.
5. Clean the diamond with isopropanol.
6. Thaw urinary EV sample and load 2 µL of the filtrate on the ATR diamond.
7. Blow dry the solution on the diamond using a blow dryer.
8. Run triplicates for each sample.
9. Save the spectrum files (.sp format)

3 . **Process acquired IR spectra and apply multivariate statistical analysis**

Materials

- Spectrum files of urinary EVs, water and PBS
- The Unscrambler X 10.5.1 software

Procedure

1. Import all the extracellular vesicles spectra into the software (.sp format).
2. Correct baseline by performing baseline correction.
3. Perform unit vector normalization on the spectra.
4. Apply derivative Savitsky−Golay transformation at the following parameters:
  - Derivative order: 1
  - Polynomial order: 2
  - Smoothing points: 21
  - Left point: 10
  - Right points: 10
5. Apply standard normal variate transformation.
6. Apply principal component analysis (PCA) to the wavelength ranged between 1794–813 cm^−1^ at the following parameters:
  - Number of principal components (PCs) included in analysis: 7
  - Validation type: cross validation
7. Generate PCA score plot using the first and second principal components (PC1 and PC2).

4 . **Establish reference matrix and prostate cancer classification**

Materials

- Urinary EVs of participants in transrectal ultrasound (TRUS)-guided prostate biopsy
- Excel/data science program e.g., Phyton for derivation of Euclidean distances

Procedure

1. Isolate EVs from urine samples collected from healthy individuals and patients diagnosed with different stages of prostate cancers (Gleason scores <7, =7 & >7).
2. Acquire and process IR spectra from all the collected EV samples.
3. Analyze all the processed spectra and generate a PCA score plot. This is the reference plot for sample analysis.
4. Export the datasets of PC1 and PC2 from Unscrambler software.
5. Import the datasets into Excel/Phyton for derivation of average points (centroids) in PCA score plots based on the mean of all samples in the respective clusters. These centroids are reference points for sample classification.
6. Collect urinary EV samples from patients suspected of prostate cancer.
7. Acquire and process IR spectra from the collected samples.
8. Add the processed sample’s spectral data to the reference PCA dataset to obtain the coordination of the sample in score plot.
9. Calculate the Euclidean distances between the sample’s point and the centroids of respective clusters.
10. Classify the sample base on minimum Euclidean distance, e.g., FTIR PCA analysis of urinary EVs shown in Figure A7 classified patient X in the cluster of Gleason score = 7
